# Triglyceride-mimetic prodrugs of scutellarin enhance oral bioavailability by promoting intestinal lymphatic transport and avoiding first-pass metabolism

**DOI:** 10.1080/10717544.2021.1960928

**Published:** 2021-08-02

**Authors:** Xinran Wang, Cai Zhang, Ning Han, Juyuan Luo, Shuofeng Zhang, Chunguo Wang, Zhanhong Jia, Shouying Du

**Affiliations:** a School of Chinese Materia Medica, Beijing University of Chinese Medicine, Beijing, China; b Beijing Research Institute of Chinese Medicine, Beijing University of Chinese Medicine, Beijing, China

**Keywords:** Scutellarin, triglyceride-mimetic prodrugs, intestinal lymphatic transport, oral bioavailability

## Abstract

The intestinal capillary pathway is the most common way to absorb oral drugs, but for drugs with poor solubility and permeability and high first-pass metabolism, this pathway is very inefficient. Although intestinal lymphatic transport of lipophilic drugs or prodrugs is a promising strategy to improve the oral delivery efficiency of these drugs. The prodrug strategy for modifying compounds with Log *P* > 5 to promote intestinal lymphatic transport is a common approach. However, transport of poor liposoluble compounds (Log *P* < 0) through intestinal lymph has not been reported. Herein, triglyceride-mimetic prodrugs of scutellarin were designed and synthesized to promote intestinal lymphatic transport and increase oral bioavailability. Lymphatic transport and pharmacokinetic experiments showed that two prodrugs did promote intestinal lymphatic transport of scutellarin and the relative oral bioavailability was 2.24- and 2.45-fold of scutellarin, respectively. In summary, triglyceride-mimetic prodrugs strategy was used for the first time to study intestinal lymphatic transport of scutellarin with Log *P* < 0, which could further broaden the application range of drugs to improve oral bioavailability with the assistance of intestinal lymphatic transport.

## 1. Introduction

**Figure 1.**
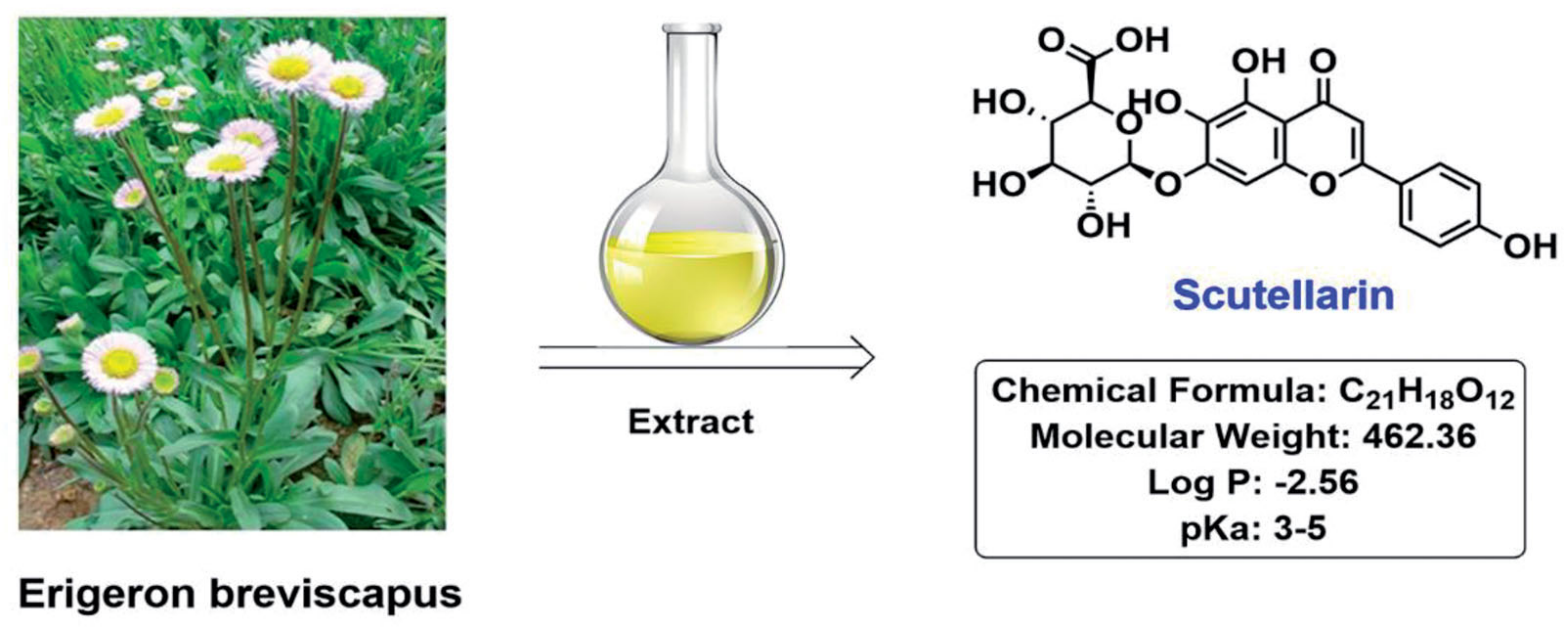
Structure of scutellarin.

Scutellarin (Log *P* = −2.56), a natural product of flavonoids, is the main active ingredient of traditional Chinese herbs *Erigeron breviscapus* (Figure 1), which has the effect of promoting blood circulation, removing blood stasis, dredging meridians, and relieving pain. Clinical drugs with scutellarin as the main active ingredient include breviscapine tablets, breviscapine injection, etc., which are widely used in the treatment of apoplexy sequelae, coronary heart disease, angina pectoris, and various ischemic cardio-cerebrovascular diseases (Cao et al., 2006; Chen et al., 2006; Alouane et al., 2015; Chledzik et al., 2018). In recent years, researchers have found that scutellarin also has anti-inflammatory, anti-oxidant, and anti-thrombotic activities (Feng et al., 2012; Dal Corso et al., 2020), and has certain curative effects on Alzheimer's disease, Helicobacter pylori infection, vascular complications of diabetes and cancers (Gao et al., 2011; Guo et al., 2013), indicating that scutellarin is a natural product with great potential.

**Figure 2.**
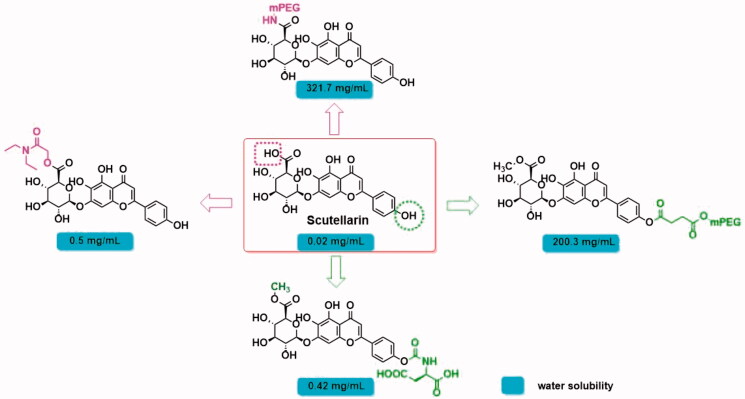
Modification of scutellarin.

Although the extensive pharmacological activity of scutellarin has attracted the attention of researchers, as a BCS class IV drug, its low solubility and low permeability lead to poor pharmacokinetic properties, which seriously hinders the further development of this compound. The oral bioavailability of scutellarin in rats and beagles was 10.6 and 0.4% (Huang et al., 2005), respectively. Plasma *C*_max_ was < 5.0 ng/mL when 60 mg of scutellarin was taken orally in humans (Lu et al., 2010). To improve its pharmaceutical properties, structural modification and dosage form optimization were tried. In terms of structural modification, the researchers introduced hydrophilic fragments into the carboxyl group or 4′-phenolic hydroxyl group of scutellarin (Marina Shamis and Shabat, 2003; Ma et al., 2015) to improve the water solubility of the molecule (Figure 2). Unfortunately, although this solution could remarkably improve the water solubility of the molecule, it did not show significant improvement in oral absorption. On the other hand, the encapsulation of scutellarin in liposomes, nanoparticles, nanoemulsion, and other dosage forms can improve the gastrointestinal stability and permeability of drugs (Mu and Høy, 2004; Peng Dayan, 2011; Ma et al., 2015; Wang et al., 2017; Markovic et al., 2020), and its relative oral bioavailability (*F*_rel_) and area under the drug-time curve (AUC) can be increased by 2- to 3-fold. However, the deficiency of low drug loading has brought inconvenience to its clinical application. How to improve the oral bioavailability of scutellarin is still an urgent problem.

**Figure 3.**
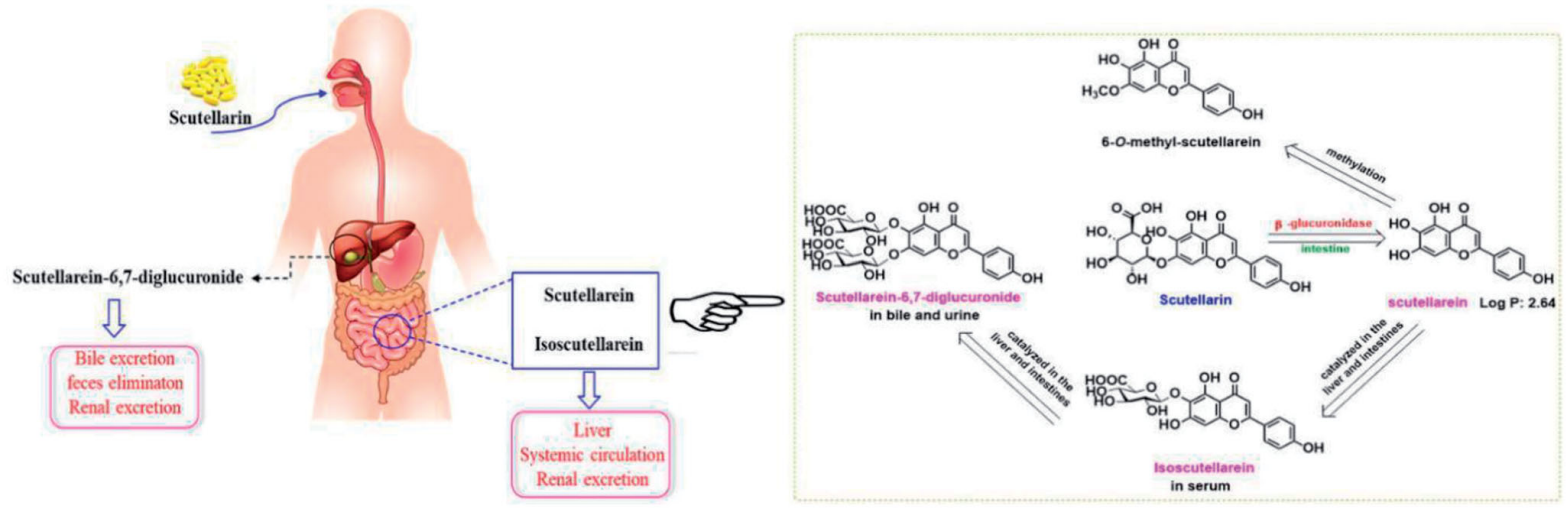
Metabolism of scutellarin.

To find a solution to this problem, we reviewed the literature on the absorption and metabolism of scutellarin (Qiu et al., 2007; Gao et al., 2011; Wang et al., 2011; Xing et al., 2011; Gao et al., 2012; Trevaskis et al., 2015; Ryšánek et al., 2020). It was found that scutellarin was difficult to penetrate intestinal epithelial cells and most of it was broken down by β-glucuronidase to generate aglycones. The generated aglycones can be absorbed through intestinal epithelial cells and metabolized before entering the portal vein (Wang et al., 2017). Further glycosylation occurs after entering the liver, which accelerates excretion (Figure 3). These studies indicate that poor membrane permeability, high intestinal capillary metabolism, and liver first-pass metabolism are the root causes of poor oral bioavailability of scutellarin. Intestinal capillary absorption pathway is difficult to guarantee an acceptable oral bioavailability of scutellarin.

**Figure 4.**
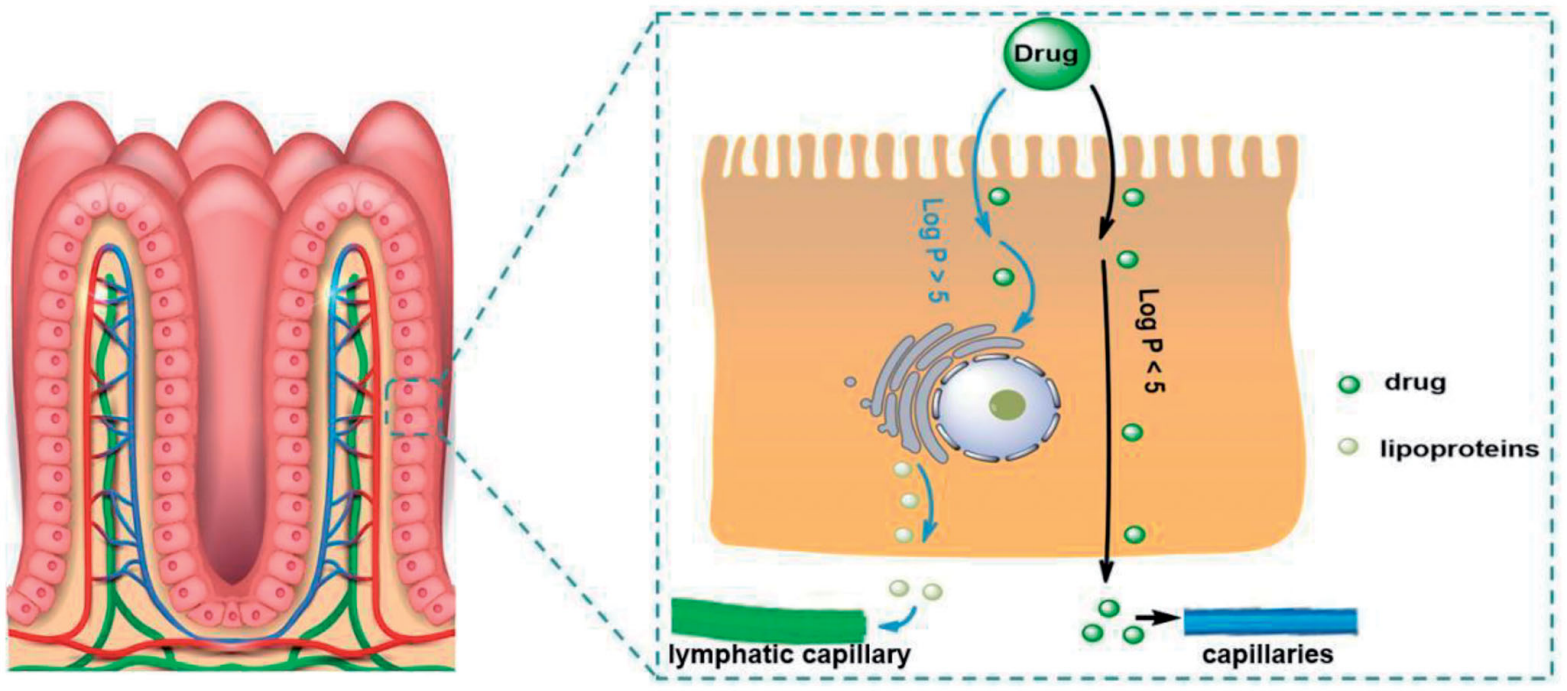
The process by which different drug molecules are absorbed by capillaries and lymphatic capillaries in the intestinal tract.

Drug delivery through intestinal lymphatic vessels, which provides a new idea for solving the problem of drug absorption with serious first-pass metabolism (Xing et al., 2011; Wang and Ma, 2018), and also points out the direction for improving the oral absorption of scutellarin. Based on the reported studies, most of the compounds studied for intestinal lymphatic transport are molecules with high liposolubility (Log *P* > 5), and increasing the liposolubility of the drug usually enhances intestinal lymphatic transport (Yan et al., 2010) (Figure 4). Many drugs have been modified into lipophilic prodrugs, such as alkyl ester prodrugs, phospholipid prodrugs, and triglyceride prodrugs, to enhance intestinal lymphatic transport. Altering the dosage form of the drug can also promote intestinal lymphatic transport. Topotecan (Log *P* = 0.8) encapsulation in core-shell lipid nanoparticles can promote intestinal lymphatic transport, demonstrating that a low-fat soluble compound can enhance intestinal lymphatic transport and promote oral absorption through dosage form optimization (Wang et al., 2017). Although the reported drugs that can be transported through intestinal lymph are all molecules with Log *P* > 0, they still provide us with reference. We proposed that it is feasible to utilize a prodrug strategy to increase the liposolubility of scutellarin (Log *P* = −2.56) to promote intestinal lymphatic transport and improve its oral absorption. 

Since the intestinal lymph is responsible for the transport of dietary lipids (primarily triglyceride, TG), this means that triglyceride-mimetic prodrugs (TG-drug) of scutellarin can not only improve lipid solubility and permeability but also simulate the absorption process of dietary triglyceride to promote the entry of prodrug molecules into the mesenteric lymphatic system (Yang et al., 2014). As with the intestinal absorption of triglycerides (Mu and Høy, 2004), Drug-TG is hydrolyzed into monoglyceride prodrugs (MG-drug) and free fatty acids (FA) in the intestinal tract, then forms micelles with bile acids and enters intestinal epithelial cells. The intracellular MG-drug and FA are re-esterified into TG-drug in the endoplasmic reticulum and the re-esterified TG-drug will be involved in the formation of lipoproteins (LP) in the Golgi apparatus, which then enters the intestinal basement membrane through exocytosis and is absorbed by the lymphatic capillaries of the intestine. The TG-drug transported through the lymphatic system is eventually absorbed into the vein, where the parent drug can be released under the action of lipoprotein lipase (LPL).

**Figure 5.**
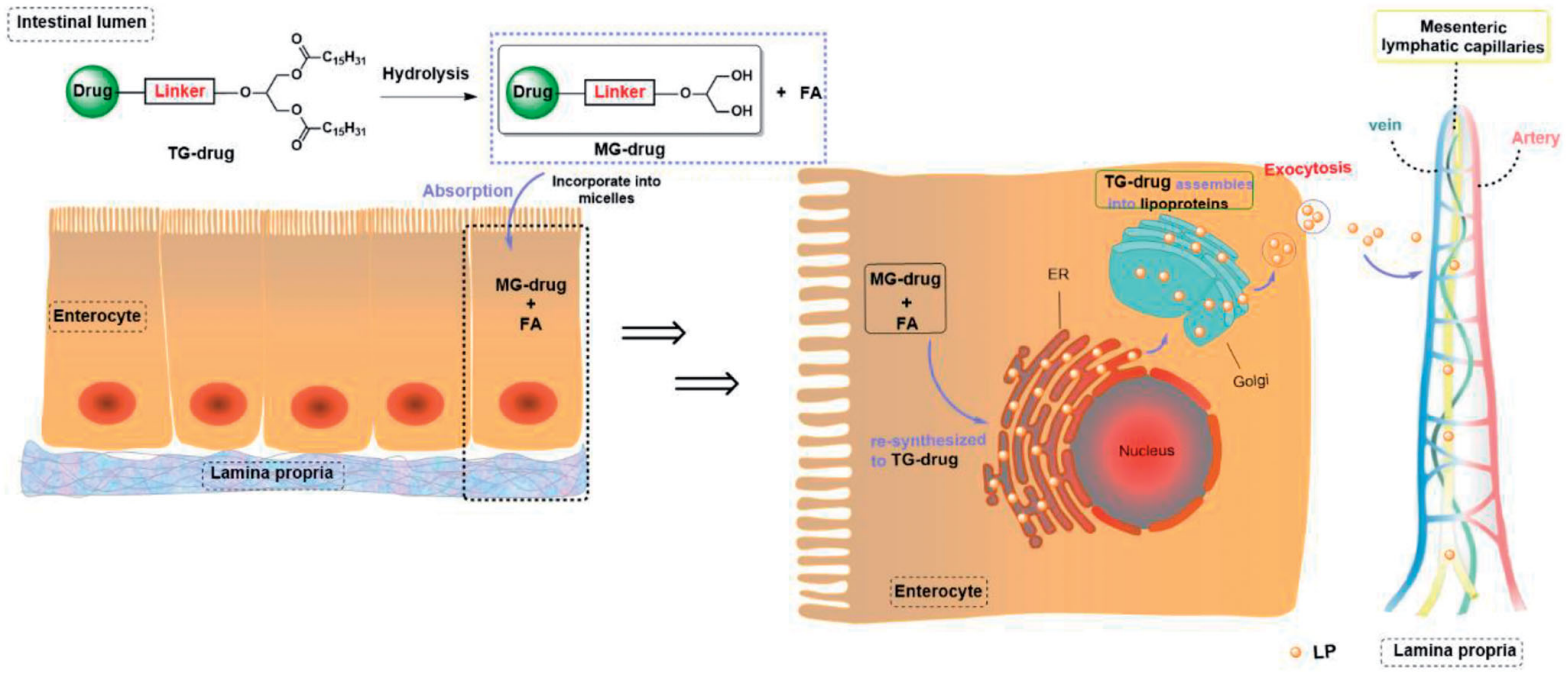
Intestinal absorption of triglyceride prodrugs after oral administration.

Herein, we engineered scutellarin as a triglyceride-mimetic prodrug to increase lymphatic transport and oral absorption (Figure 5). Simulated gastric fluid (SGF), simulated intestinal fluid (SIF), and rat plasma were used to evaluate scutellarin triglyceride-mimetic prodrug *in vitro*. To assess the lymphatic transport of the designed prodrug, the UHPLC-MS/MS technique was used for quantification and oral bioavailability was determined. To our knowledge, this is the first study to report intestinal lymphatic transport of a molecule with Log *P* < 0 using a triglyceride-mimetic prodrug strategy and the intestinal lymphatic transport of scutellarin (Log *P* = −2.56) was studied for the first time.

## 2. Materials and methods

### 2.1. Materials and reagent

1,3-Dipalmitoylglycerol was purchased from Bachem, Bubendorf, Switzerland. Scutellarin, dihydro-2*H*-pyran-2,6(3*H*)-dione, 1-(3-dimethylaminopropyl)-3-ethyl carbodiimide hydrochloride (EDCI), 4-dimethylaminopyridine (DMAP), 4-methyl dihydro-2*H*-pyran-2,6(3*H*)-dione were obtained from Bide Pharmaceutical Technology Co., Ltd., Shanghai, China. All organic reagents and solvents used for synthesis were the analytical pure grade. TLC silica gel plates and silica gel for column-layer chromatography (300–400 mesh) were purchased from Qingdao Haiwan Specialty Chemicals Co., Ltd., Qingdao, China. Simulated gastric fluid, simulated intestinal fluid, and rat plasma were obtained from Yuanye Bio-Technology Co., Ltd., Shanghai, China. Female S-D rats were purchased Beijing Vital River Laboratory Animal Technology Co., Ltd., China.

### 2.2. Design of prodrugs

**Figure 6.**
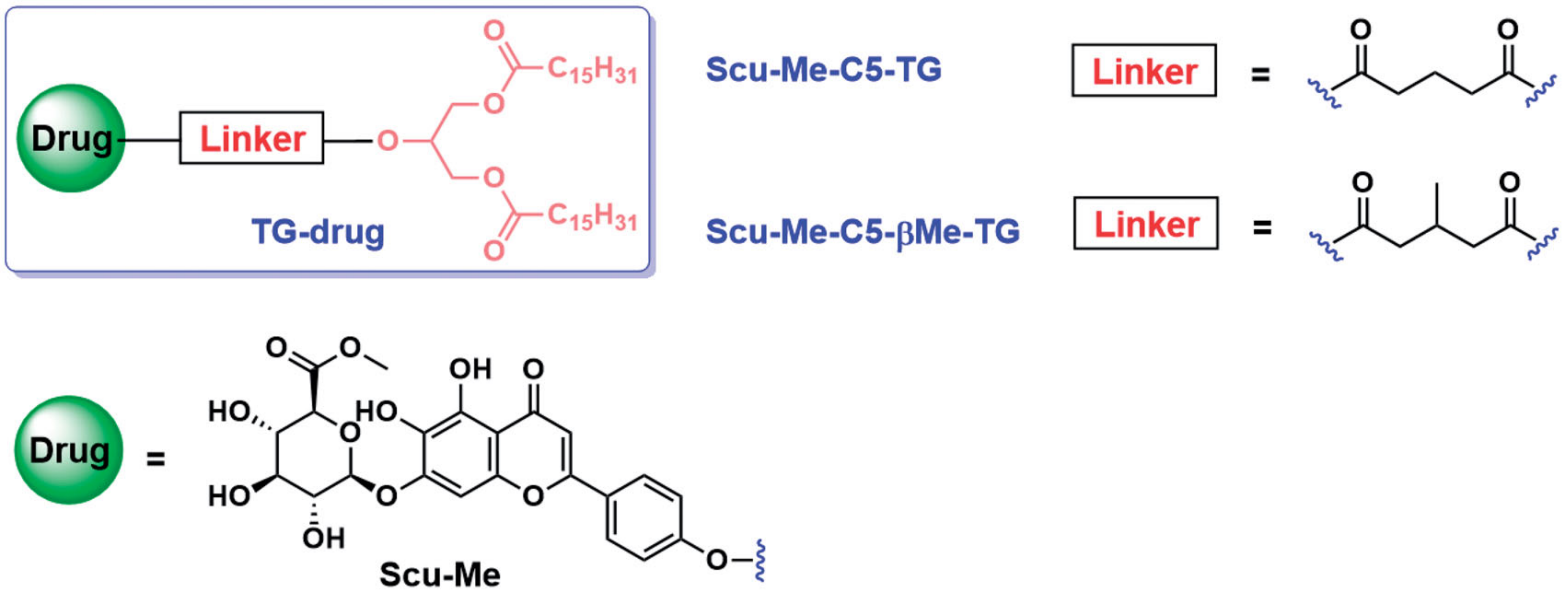
Prodrugs of scutellarin and structure of linker fragment.

Aim to increase the intestinal lymphatic transport of scutellarin, we designed two triglyceride-mimetic prodrugs of scutellarin (Scu-TG). To prevent the carboxyl group from dissociating in a weakly alkaline intestinal environment, we masked the carboxyl group in scutellarin by an ester bond (Scu-Me). Then, a C5 linker was selected to link scutellarin moiety with diglyceride (DG) through ester linkage to obtain produg 1 (Scu-Me-C5-TG, Figure 6). As triglyceride prodrugs have the potential to be completely hydrolyzed to glycerol and fatty acids in the small intestine, so we introduced methyl at the C5 linker to increase steric hindrance and prevent further hydrolysis of the monoglycerol, and obtained prodrug 2 (Scu-Me-C5-βMe-TG).

### 2.3. Synthesis of prodrugs

**Scheme 1.**
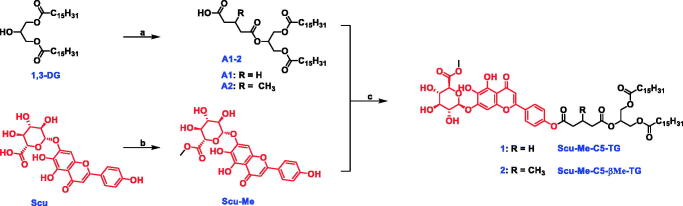
Synthetic pathway for prodrugs. (a) Corresponding anhydride, DMAP, DCM, r.t., 24 h, 46.3–50.3%; (b) SOCl_2_, MeOH, 0 °C, overnight, 82.5%; (c) EDCI, DMAP, DMF, r.t., overnight, 25.6–33.5%.

Prodrugs were synthesized by bridging Scu-Me and glyceride moieties with a C5 linker (Scheme 1). Scu-Me was obtained by esterification of scutellarin with methanol in the presence of sulfoxide chloride. The intermediate A was obtained by acylation of 2-hydroxypropane-1,3-diyl dipalmitate (1,3-DG) with the corresponding anhydride. The intermediate A was then condensed with Scu-Me to obtain the prodrugs.

#### 2.3.1. Synthesis of 5-((1,3-bis(palmitoyloxy)propan-2-yl)oxy)-5-oxopentanoic acid (A1)

1,3-DG (0.50 g, 0.88 mmol) was dissolved in 10 mL dried dichloromethane, then dihydro-2*H*-pyran-2,6(3*H*)-dione (0.20 g, 1.76 mmol) and 4-dimethylamino pyridine (0.16 g, 1.32 mmol) were added. The mixture was stirred at room temperature for 24 h, and then 50 mL dichloromethane was added to the reaction solution and the resulting solution was washed with dilute hydrochloric acid solution (3 × 10 mL). The dichloromethane layer was then washed with saturated salt solution (3 × 10 mL) and dried with anhydrous sodium sulfate and concentrated. Chromatography on silica gel (dichloromethane: methanol = 50: 1) to afford A1 (0.30 g, 56.7%) as a white solid. MS (ESI): C_40_H_74_O_8_, [M − H]^−^: 681.53. ^1^H NMR (400 MHz, CDCl_3_) *δ* 5.30–5.23 (m, 1H), 4.33 (d, *J* = 4.3 Hz, 1H), 4.30 (d, *J* = 4.3 Hz, 1H), 4.16 (d, *J* = 5.9 Hz, 1H), 4.13 (d, *J* = 5.9 Hz, 1H), 2.44 (q, *J* = 7.4 Hz, 4H), 2.31 (t, *J* = 7.6 Hz, 4H), 1.97 (p, *J* = 7.3 Hz, 2H), 1.64–1.57 (m, 4H), 1.25 (s, 48H), 0.88 (t, *J* = 6.8 Hz, 6H).

The intermediate A2 was prepared by the same method with a yield of 46.3%. MS (ESI): C_41_H_76_O_8_, [M − H]^−^: 695.55. ^1^H NMR (400 MHz, CDCl_3_) *δ* 5.31–5.25 (m, 1H), 4.33 (dd, *J* = 4.2, 2.8 Hz, 1H), 4.30 (dd, *J* = 4.1, 2.8 Hz, 1H), 4.16 (d, *J* = 6.0 Hz, 1H), 4.13 (d, *J* = 6.0 Hz, 1H), 2.51–2.40 (m, 3H), 2.31 (t, *J* = 7.5 Hz, 6H), 1.64–1.57 (m, 4H), 1.27 (d, *J* = 9.2 Hz, 48H), 1.07 (d, *J* = 6.3 Hz, 3H), 0.88 (t, *J* = 6.8 Hz, 6H).

#### 2.3.2. Synthesis of methyl (2S,3S,4S,5R,6S)-6-((5,6-dihydroxy-2-(4-hydroxyphenyl) -4-oxo-4H-chromen-7-yl)oxy)-3,4,5-trihydroxytetrahydro-2H-pyran-2-carboxylate (Scu-Me)

Sixty milliliters of anhydrous methanol was added to the reaction flask and cooled in an ice bath. Then sulfoxide chloride (2 mL) was added drop by drop and stirred another 1 h after addition. Scutellarin (1.00 g, 2.16 mmol) was added to the reaction mixture and then gradually restored to room temperature, where it was stirred for 24 h. The reaction solution was filtered and washed twice with a small amount of cold methanol. The obtained filter cake was dried and the product was obtained as a yellow solid (0.85 g, 82.5%). MS (ESI): C_22_H_20_O_12_, [M − H]^−^: 475.09. ^1^H NMR (400 MHz, DMSO) *δ* 12.74 (s, 1H), 10.35 (s, 1H), 8.59 (s, 1H), 7.93 (d, *J* = 8.8 Hz, 2H), 7.00 (s, 1H), 6.95 (d, *J* = 8.8 Hz, 2H), 6.81 (s, 1H), 5.49 (s, 2H), 5.27 (d, *J* = 7.4 Hz, 1H), 4.20 (d, *J* = 9.5 Hz, 1H), 3.68 (s, 3H), 3.47–3.34 (m, 4H).

#### 2.3.3. Synthesis of 1,3-bis(palmitoyloxy)propan-2-yl (4-(5,6-dihydroxy-4-oxo-7- (((2S,3R,4S,5S,6S)-3,4,5-trihydroxy-6-(methoxycarbonyl)tetrahydro-2H-pyran-2-yl)oxy)-4H-chromen-2-yl)phenyl) glutarate (Scu-Me-C5-TG)

A1 (0.30 g, 0.44 mmol), Scu-Me (0.25 g. 0.53 mmol), EDCI (0.25 g, 1.32 mmol), and DMAP (0.08 g, 0.66 mmol) were dissolved in 4 mL anhydrous *N,N*-dimethylformamide and stirred for 48 h at room temperature. The reaction solution was poured into 100 mL of water and adjusted to pH = 4 with dilute hydrochloric acid. The mixture was filtered and filter cake was purified by silica gel column chromatography to obtain Scu-C5-TG as yellow solid (0.17 g, 33.5%). HR-MS (ESI): C_62_H_92_O_19_, [M + Na] ^+^: 1163.6122. ^1^H NMR (400 MHz, CDCl_3_) *δ* 12.92 (s, 1H), 7.60 (d, *J* = 8.3 Hz, 2H), 6.78 (d, *J* = 8.0 Hz, 2H), 6.60 (s, 1H), 6.46 (s, 1H), 5.34–5.27 (m, 1H), 5.10 (d, *J* = 6.3 Hz, 1H), 4.40–4.33 (m, 2H), 4.26–4.17 (m, 3H), 4.08 (s, 1H), 3.95–3.88 (m, 1H), 3.84 (s, 3H), 3.77 (d, *J* = 5.1 Hz, 2H), 3.48 (d, J = 25.3 Hz, 2H), 2.79–2.74 (m, 2H), 2.58 (t, *J* = 7.1 Hz, 2H), 2.34 (td, *J* = 7.6, 3.0 Hz, 4H), 2.18–2.10 (m, 2H), 1.62 (m, 8H), 1.23 (s, 48H), 0.87 (t, *J* = 6.9 Hz, 6H). ^13 ^C NMR (151 MHz, CDCl_3_) *δ* 182.28, 173.77, 173.72, 172.78, 171.75, 169.08, 164.46, 159.77, 154.15, 152.90, 128.00, 123.52, 122.31, 116.06, 107.01, 103.56, 100.85, 94.10, 75.50, 74.74, 72.69, 71.06, 69.78, 61.96, 53.07, 34.08, 33.08, 32.65, 31.92, 29.70, 29.67, 29.66, 29.63, 29.50, 29.36, 29.28, 29.13, 24.86, 22.69, 20.11, 14.12.

The Scu-Me-C5-βMe-TG was obtained by condensation of A2 and Scu-Me under the same conditions, yellow solid, yield 25.6%. HR-MS (ESI): C_62_H_92_O_19_, [M − H]^−^: 1153.6331. ^1^H NMR (400 MHz, CDCl_3_) *δ* 12.88 (d, *J* = 3.8 Hz, 1H), 7.55 (d, *J* = 8.1 Hz, 2H), 7.07 (s, 1H), 6.75 (d, *J* = 7.9 Hz, 2H), 6.58 (d, *J* = 5.7 Hz, 1H), 6.42 (s, 1H), 5.35–5.28 (m, 1H), 5.12 (d, *J* = 6.8 Hz, 1H), 4.42–4.33 (m, 2H), 4.25–4.20 (m, 3H), 3.92 (t, *J* = 8.5 Hz, 1H), 3.82 (s, 3H), 3.77 (s, 2H), 3.68 (s, 1H), 2.79–2.58 (m, 4H), 2.47 (dd, *J* = 16.1, 7.6 Hz, 1H), 2.36–2.30 (m, 4H), 1.80 (s, 2H), 1.60 (s, 4H), 1.23 (s, 48H), 1.18 (t, *J* = 6.2 Hz, 3H), 0.87 (t, *J* = 6.8 Hz, 6H). ^13 ^C NMR (151 MHz, CDCl_3_) *δ* 182.24, 173.88, 173.82, 173.78, 173.71, 172.49, 172.23, 171.14, 169.11, 164.46, 159.95, 154.13, 152.85, 127.94, 123.43, 122.08, 116.05, 106.93, 106.90, 103.39, 100.77, 100.68, 75.49, 74.84, 72.75, 71.13, 69.73, 69.62, 62.04, 62.01, 53.07, 40.52, 40.40, 40.16, 40.06, 34.10, 31.93, 29.71, 29.69, 29.67, 29.65, 29.52, 29.37, 29.29, 29.15, 27.77, 27.57, 24.87, 22.70, 20.01, 19.91, 14.13. 

### 2.4. *In vitro* stability of prodrugs

#### 2.4.1. Investigation on the stability of prodrugs in SGF and SIF

Add 16.4 mL of dilute hydrochloric acid into 800 mL of water, then add 10 g of pepsin. After mixing, add water to the total volume of 1000 mL to get simulated gastric fluid. Add 20 μL prodrug solution (1 mg/mL) into 1 mL of SGF and shake it slightly at 37 °C. 20 μL mixed solution was added into 180 μL methanol at 0, 5, 10, 15, 30, 60, 90, 120, and 180 min, respectively. The mixture was vortexed for 5 min and centrifuged at 16,000 rpm for 15 min. The supernatant was sampled and used for UHPLC-MS/MS detection.

6.8 g of potassium dihydrogen phosphate was weighed, dissolved in 500 mL of water, and the pH was adjusted to 6.8 with 0.1 mol/L sodium hydroxide solution. In addition, 10 g of trypsin was taken and dissolved in an appropriate amount of water. The two solutions were mixed, and the total volume was fixed to 1000 mL with water. The SIF was obtained after mixing well. The stability of prodrugs in SIF was determined using the same procedure as the SGF stability assessment.

#### 2.4.2. Investigation on the stability of prodrugs in rat plasma

Ten microliters lipoprotein lipase solution (10,000 IU/mL) and 10 μL prodrugs solution were added into 480 μL blank S-D rat plasma, and the mixture was incubated at 37 °C. At the same sampling time points as the stability test of SGF, 20 μL mixed solution was added into 180 μL methanol, vortexed for 5 min, and centrifuged at 16,000 rpm for 15 min. The supernatant was sampled and used for UHPLC-MS/MS detection.

### 2.5. *In vivo* lymphatic transport studies in anesthetized rats

Female Sprague Dawley rats were selected to establish the model of mesenteric lymphatic intubation and jugular vein intubation in anesthetized rats, and the drug absorption through intestinal lymphatic circulation was quantified. Briefly, the rats were fasted for 12 h before operation and divided into three groups (*n* = 5 each group) randomly: scutellarin solution (25 mg/kg), Scu-Me-C5-TG solution (62.5 mg/kg equivalent to Scu), Scu-Me-C5-βMe-TG solution (62.5 mg/kg equivalent to Scu), then the mesenteric lymphatic vessels were filled by intragastric administration of 2 mL peanut oil. After the mesenteric lymphatic vessels were filled, ∼2 h after the administration of peanut oil, scutellarin or prodrugs dissolved in peanut oil were given by intragastric administration. Subsequently, the rat was anesthetized by intraperitoneal injection of 2% pentobarbital sodium and a transverse incision was made in the middle of the abdominal cavity. The mesenteric artery was found around the right renal artery, and milky white mesenteric lymphatic vessels parallel to it were visible. The mesenteric lymphatic vessels were obtusely separated and then inserted into a silicone tube with an external diameter of 0.9 mm for ∼0.3 cm. While a centrifuge tube containing EDTA anticoagulant was inserted at the other end of the silicone tube. Centrifugal tubes were replaced every hour and collected continuously for 12 h. During the whole operation, the rats were administered with Lactated Ringer's solution via jugular vein intubation to maintain fluid balance and pentobarbital sodium solution to maintain anesthetic status. The collected lymphatic fluid was added to a 4-fold volume of methanol, and the supernatant was centrifuged after eddy and ultrasonic treatment for detection. Since the prodrugs undergo hydrolysis and re-esterification before entering the intestinal lymphatic vessels, which results in a change in molecular weight of Scu-TG in the lymph. Therefore, we hydrolyze the ester bond with 0.1 mol/L sodium hydroxide solution, and then adjust the pH with 0.2 mol/L hydrochloric acid solution, extracted the active compound with a 4-fold volume of methanol, and then re-dissolved with methanol after nitrogen blowing volatilization solvent for detection.

### 2.6. Oral bioavailability studies

Scutellarin exposure after oral administration of scutellarin and prodrugs was detected in conscious female S-D rats. The rats were fasted for 12 h before the experiment and divided into three groups (*n* = 5 each group) randomly: scutellarin solution (25 mg/kg), Scu-Me-C5-TG solution (62.5 mg/kg equivalent to Scu), Scu-Me-C5-βMe-TG solution (62.5 mg/kg equivalent to Scu). Blood samples (0.5 mL) were collected from 5 min before administration to up to 24 h post-dosing and centrifuged at 16,000 rpm for 15 min to separate plasma. 100 μL plasma was added into 900 μL methanol and vortexed for 5 min, then centrifuged at 16,000 rpm for 15 min. The supernatant was sampled and used for UHPLC-MS/MS detection. The pharmacokinetic parameters were calculated by DAS 2.0 software.

### 2.7. Statistical analysis

All data were expressed as means ± S.D. Statistical comparison was performed either with one-way ANOVA or with Student’s *t*-test (for two-group comparisons) to determine significant differences. SPSS version 25.0 (SPSS, Chicago, IL, USA) was used for data processing and GraphPad Prism 9.0 software (GraphPad, San Diego, CA, USA) was used for graphics. *p* < .05 was considered statistically significant.

## 3. Results and discussions

### 3.1. Stability studies

#### 3.1.1. Stability in SGF

**Figure 7.**
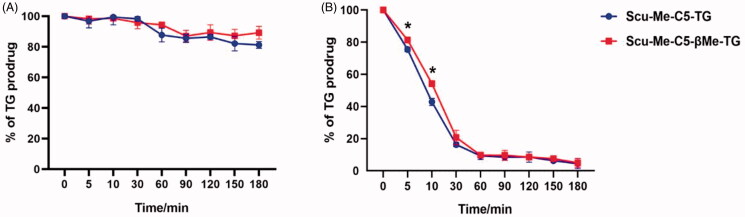
Stability of TG-drug. (A) Chemical stability of TG-drug in simulated gastric fluid; (B) Chemical stability of TG-drug in simulated intestinal fluid, *p < .05. (data are represented as mean ± *SD*, *n* ≥ 3).

As shown in Figure 7(A), the two prodrugs showed high chemical stability in SGF over 3 h. The gastric juice was analyzed by mass spectrometry and no scutellarin was detected, suggesting that the prodrugs may not release the active parent drug during the gastric emptied period. 

#### 3.1.2. Stability in SIF

The small intestine, as the main site of dietary lipids absorption, can efficiently catalyze the breakage of lipids into fatty acids and esters. The lytic products enter the intestinal epithelial cells, where they are subsequently re-esterified and enter the intestinal lymphatic capillaries. Rapid hydrolysis of triglycerides in the small intestine is the first step in the absorption process. As shown in Figure 7(B), both prodrugs could complete 80% hydrolysis in 30 min to obtain the MG-drug. Since the monoesters of glycerol are more likely to undergo enzymatic reactions to decompose into fatty acids and glycerol, we introduced methyl groups into C5-linker to prevent this process from occurring. To our disappointment, the introduction of methyl groups into linkers prevented the over-hydrolysis of prodrug molecules to some extent but did not show a significant advantage. It is possible to solve this problem by introducing groups with greater steric hindrance into C5-linker.

#### 3.1.3. Stability in rat plasma

**Figure 8.**
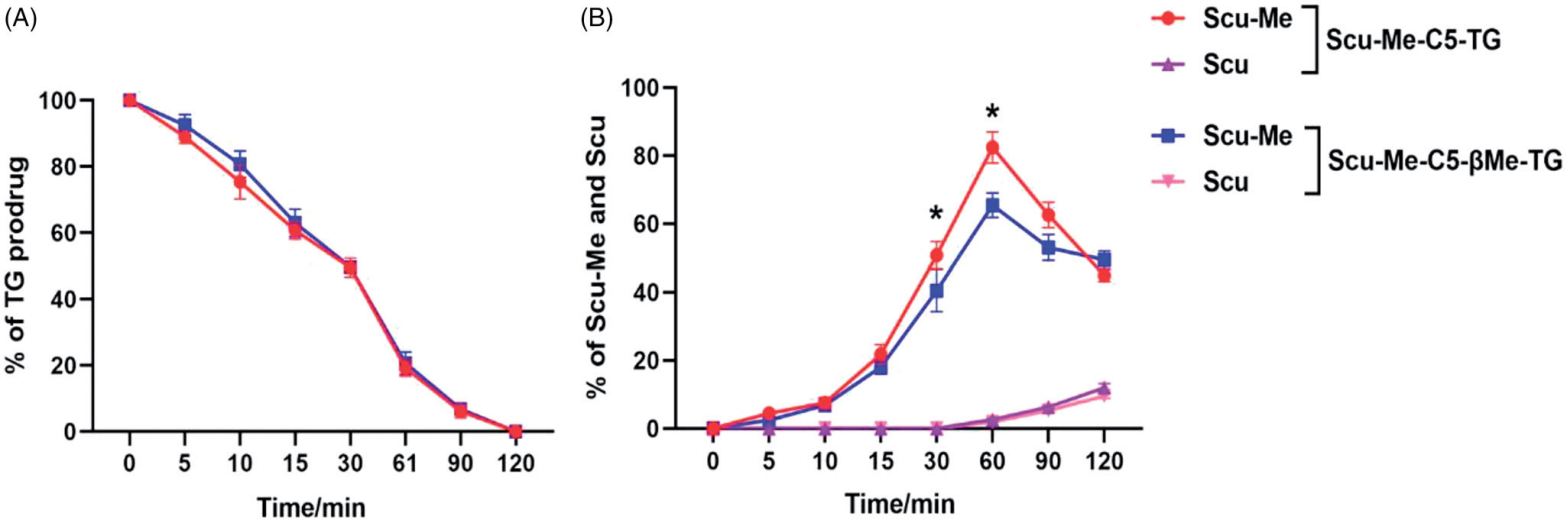
Chemical stability of TG-drug in rat plasma supplying with lipoprotein lipase. (A) The enzymatic hydrolysis rate of TG-drug in rat plasma supplying with lipoprotein lipase; (B) Production of Scu-Me and Scu of the TG-drug upon *in vitro* incubation with rat plasma supplying with lipoprotein lipase, *p <.05. (data are represented as mean ± *SD*, *n* ≥ 3).

*In vitro* plasma stability tests can be used to predict the stability of prodrugs after they enter the bloodstream and to predict the process of release of the parent drug. LPL is an important hydrolytic enzyme in vascular endothelium, which is responsible for the hydrolysis of the ester bonds in prodrug molecules to release the parent drug. Therefore, we added LPL in rat plasma to simulate the *in vivo* environment. The results (Figure 8) showed that the ester bond in C5-linker of the two TG-drug could fracture 50% within 30 min and cleavage completely within 2 h. In the initial 1 h, the TG-drug were first cleavaged to Scu-Me rather than scutellarin, and with prolonged incubation, the Scu-Me continued to hydrolyze methyl to produce scutellarin. This process of drug release may be beneficial for the improvement of bioavailability because Scu-Me can bind to the protein more easily than scutellarin to achieve progressive release of the drug to maintain the blood concentration of scutellarin.

### 3.2. Lymphatic transport studies

**Figure 9.**
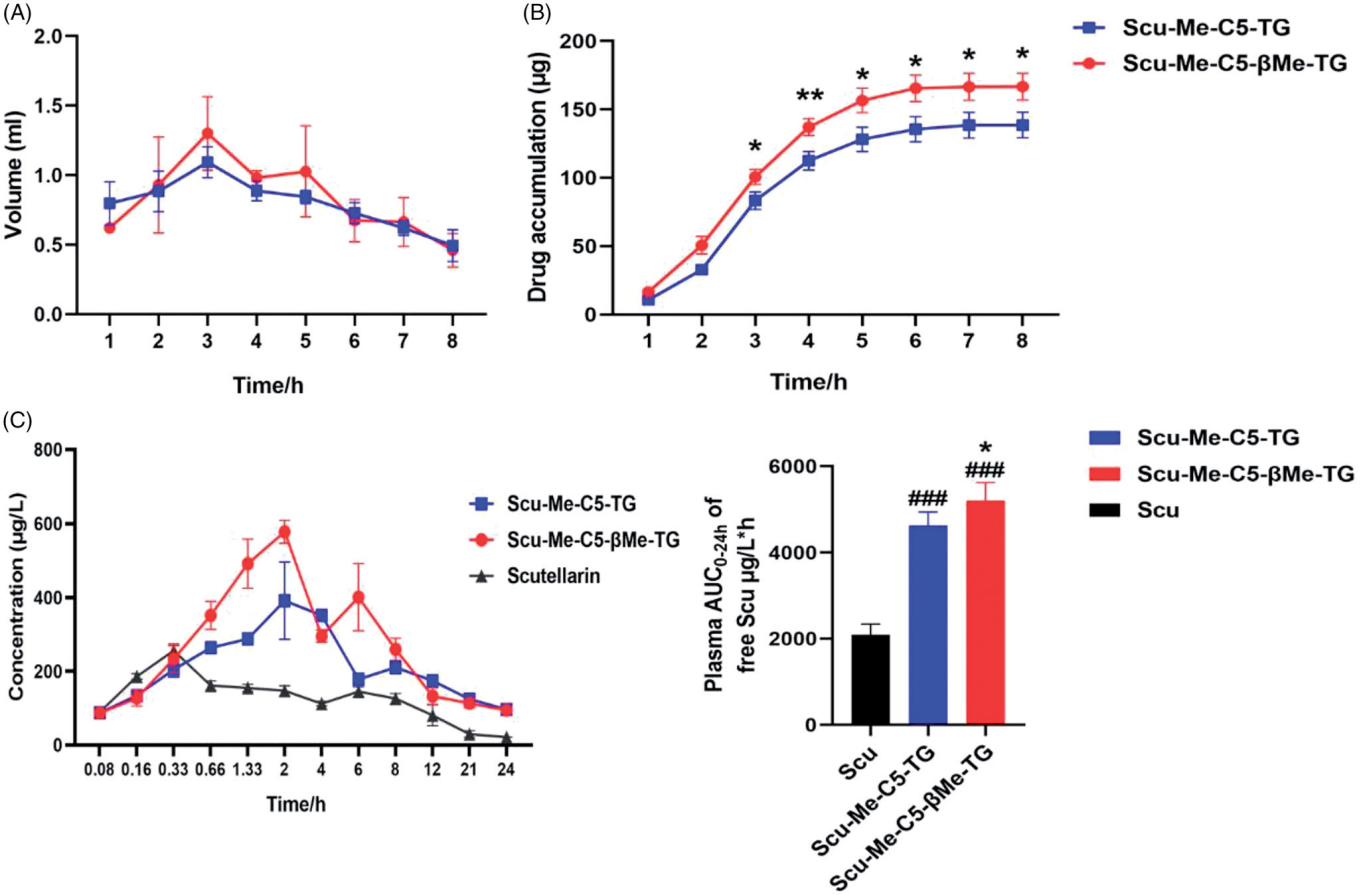
Lymphatic absorption of TG-drug. (A) The volume of lymph collected per hour after intragastric administration in female rats; (B) Cumulative lymphatic transport of Scu-Me-C5-TG and Scu-Me-C5-βMe-TG following intragastric administration to anesthetized mesenteric lymphduct-cannulated female rats; (C) Plasma concentrations of free Scu *vs.* time following oral administration of Scu (25 mg/kg), Scu-Me-C5-TG (62.5 mg/kg equivalent dose of Scu), and Scu-Me-C5-βMe-TG (62.5 mg/kg equivalent dose of Scu) to female rats. *p < .05, **p < .01, *^###^p* < .001. (data are represented as mean ± *SD*, *n* ≥ 3).

To intuitively reflect the intestinal lymphatic transport of scutellarin and its TG-drug, female S-D rats were selected to establish a mesenteric lymphatic intubation model, and the drug was quantitatively detected after lymph collection. According to the published literature (Hu et al., 2016; Tian et al., 2019), the data obtained from the model of anesthetized rats are very similar to the experimental results of conscious rats. Therefore, the anesthesia model was selected to ensure the success rate of the experiment. During the experiment, it was found that all rats could successfully collect lymph for 8 h, so the first 8 h of lymph were analyzed (Figure 9). Since the lymph collected by the scutellarin administration group failed to reach the limit of quantification in mass spectrometry, we assumed that scutellarin would not be absorbed through the lymphatic capillaries of the intestine during oral administration. Therefore, we did not present the relevant data of the scutellarin administration group here.

In the case of timely replenishment of lactate Ringer's solution, each model rat was able to collect ∼6 mL of lymph, of which both the volume and drug concentration of lymph reached their peak at the third hour after intragastric administration, and the drug concentration began to decline 3 h later. Within 8 h after administration, Scu-Me-C5-TG and Scu-Me-C5-βMe-TG were absorbed 138.6 and 166.7 μg, respectively, accounting for 2.8 and 3.3% of the dose, confirming that Scu-TG prodrug could promote the intestinal lymphatic transport of scutellarin.

### 3.3. Oral pharmacokinetic studies

Next, the oral bioavailability of the two prodrugs was tested, and the results were shown in Figure 9(C). According to the experimental data (Table 1), the AUC_(0-24h)_ of Scu-Me-C5-TG and Scu-Me-C5-βMe-TG were 4697.6 and 5146.4 μg/L*h, respectively, and the relative oral bioavailability (*F*_rel_) was 2.24- and 2.45-fold of scutellarin, respectively. At the same time, the maximum plasma concentrations(*C*_max_) of the two prodrugs were also higher than scutellarin, which were 1.72- and 2.11-fold of the parent drug, respectively. Scu-Me-C5-βMe-TG showed slightly better plasma exposure, which may be attributed to linker modification, and further structural modification of the linker may obtain better in vivo data. Furthermore, the prodrug strategy could delay the peak time of oral absorption, and the tmax of prodrug 1 was 2.8 h. 

**Table 1.** Pharmacokinetics parameters for scutellarin, Scu-Me-C5-TG and Scu-Me-C5-βMe-TG after oral administration in female rats (data are represented as mean ± SD, *n* = 5).

Parameters	Units	Scu	Scu-Me-C5-TG^a^	Scu-Me-C5-βMe-TG^b^^,c^
AUC_(0–24 h)_	μg/L*h	2098.6 ± 245.4	4697.6 ± 311.7	5146.4 ± 505.7
*t*_max_	h	0.32 ± 0.02	2.80 ± 1.09	2.40 ± 0.89
*C*_max_	μg/L	256.3 ± 18.0	441.8 ± 101.9	540.8 ± 108.9
*t*_1/2_	h	8.6 ± 0.9	10.6 ± 1.9	6.5 ± 0.9
*F*_rel_	%	100.0	223.8	245.2

^a^The *p*-value between Scu and Scu-Me-C5-TG is <.001.

^b^The *p*-value between Scu and Scu-Me-C5-βMe-TG is <.001.

^c^The *p*-value between Scu-Me-C5-TG and Scu-Me-C5-βMe-TG is <.05.

## 4. Conclusions

Glyceride-mimetic prodrugs strategy was widely used to promote intestinal lymphatic transport and enhance the oral bioavailability of highly lipid-soluble molecules. For the first time, we apply this strategy to scutellarin (Log *P* = −2.56), a molecule with poor liposolubility, confirmed that Scu-TG prodrug can promote drug absorption through the intestine lymphatic capillaries and improve oral bioavailability. This suggests that improving the oral bioavailability of compounds by promoting intestinal lymphatic transport is also applicable to the compounds with poor liposolubility. Although < 5% of the scutellarin was absorbed by the intestinal lymph, the improvement in oral bioavailability of the drugs was significant. The TG derivative was expected to be more lipid-soluble, assisting in formulation preparation. Hence, in combination with pharmaceutical technology, Scu-TG prodrug may change the current situation that scutellarin is mainly administered by intravenous injection. Further prodrug design of scutellarin to increase intestinal lymphatic transport is worthy of investigation, and it also provides new possibilities for improving the oral bioavailability of BCS class IV active ingredients in traditional Chinese herbs and other poorly lipid-soluble compounds. In our future studies, the mechanism of absorption and transport of prodrugs will be studied applying the cell and tissue model of intestinal absorption. And how prodrugs are metabolized to release Scu will be explored.

## Supplementary Material

Supplemental Material
