# Supplementary material for: Triglyceride-mimetic prodrugs of scutellarin enhance oral bioavailability by promoting intestinal lymphatic transport and avoiding first-pass metabolism
Source: Drug Deliv. 2021 Aug 2;28(1):1664–72. doi: 10.1080/10717544.2021.1960928 (PMC8330727; doi:10.1080/10717544.2021.1960928)
Supplement: Supplemental Material [file IDRD_A_1960928_SM7197.docx]

**Triglyceride-mimetic prodrugs of scutellarin enhance oral bioavailability by promoting intestinal lymphatic transport and avoiding first-pass metabolism**

Xinran Wang ^a^, Cai Zhang ^b^, Ning Han ^a^, Juyuan Luo ^a^, Shuofeng Zhang ^a^, Chunguo Wang ^b^, Zhanhong Jia ^a^, Shouying Du ^a,^*

^a^ School of Chinese Materia Medica, Beijing University of Chinese Medicine, Chaoyang District, Beijing 102488, China

^b^ Beijing Research Institute of Chinese Medicine, Beijing University of Chinese Medicine, Chaoyang District, Beijing 102488, China

^*^ Correspondence: dsy_bucm@163.com; Tel.: +86-010-53912124

1. **Testing apparatus and conditions**

The NMR experiments were performed on Bruker 400 MHz and 600 MHz instruments using TMS as an internal standard and CDCl_3_ or DMSO-*d_6_* as solvent. The in vitro stability of the compounds was detected by ultrahigh-pressure liquid chromatography (UHPLC), which was performed on a Thermo Scientific™ Vanquish™ Flex UHPLC systems (Thermo Scientific, Santa Clara, CA, USA). Samples or strands were separated on an AQUITY UPLC @HSS T3 column (2.1 mm*100 mm, 1.8 μm) at 40°C. The gradient eluent conditions were as follows: 0-2 min (20% A), 2-6 min (40% A), 6-9 min (60% A), 9-15 min (95% A), 15-42 min (95% A), 42-50 min (20% A), in which A is acetonitrile and B is 0.1% formic acid aqueous solution. The injection volume was 0.3 μL, and the flow rate was 0.3 mL/min. The peaks were detected at λ = 338 nm. Qualitative and quantitative mass spectrometric analysis was performed on the UHPLC-LTQ-Orbitrap MS instrument platform (Thermo Scientific, Santa Clara, CA, USA). The internal standard substance used in liquid and mass spectrometric tests was [wogonoside](https://www.chemsrc.com/en/cas/51059-44-0_670611.html).

1. **The mass spectra, ^1^H-NMR and ^13^C-NMR spectra of the intermediates and the target products**

**Figure S1.** Mass spectra of A1


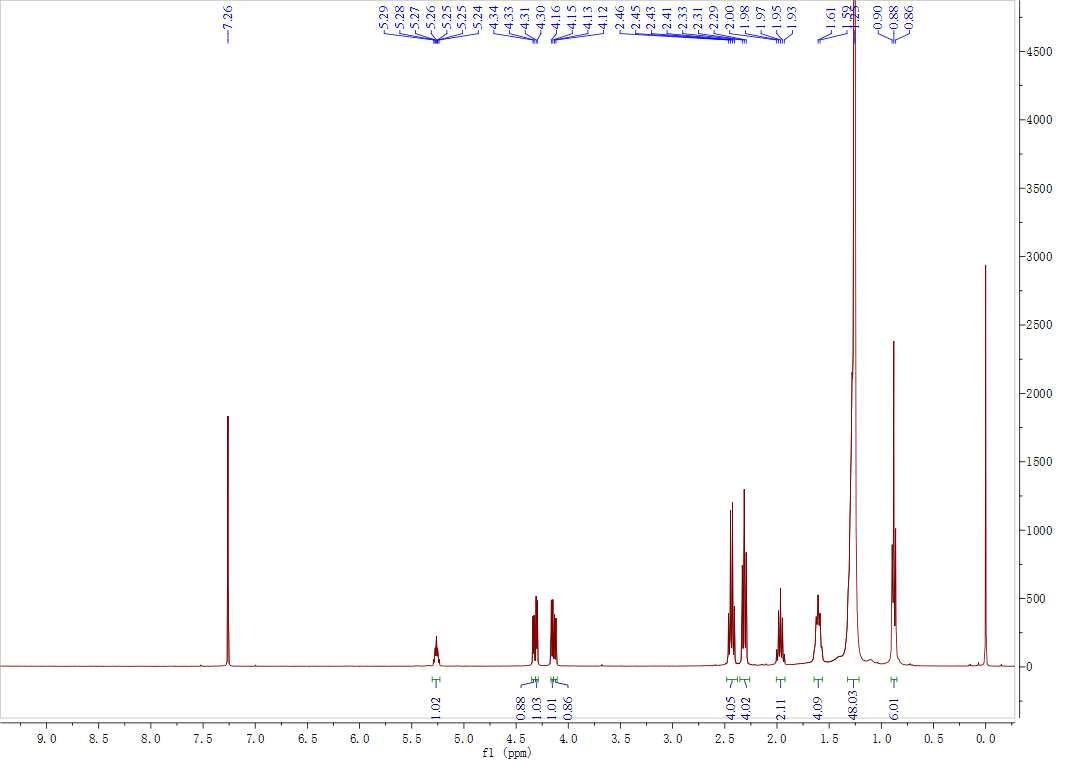


**Figure S2.** ^1^H-NMR spectra of A1

**Figure S3.** Mass spectra of A2


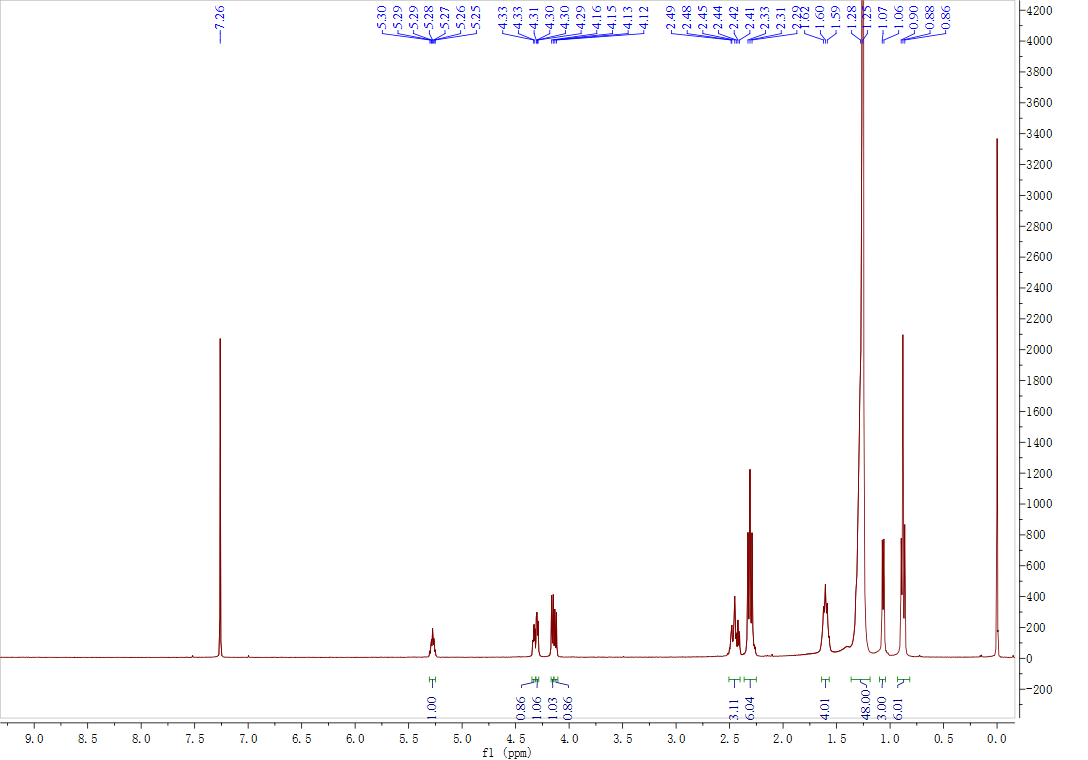


**Figure S4.** ^1^H-NMR spectra of A2

**Figure S5.** Mass spectra of Scu-Me


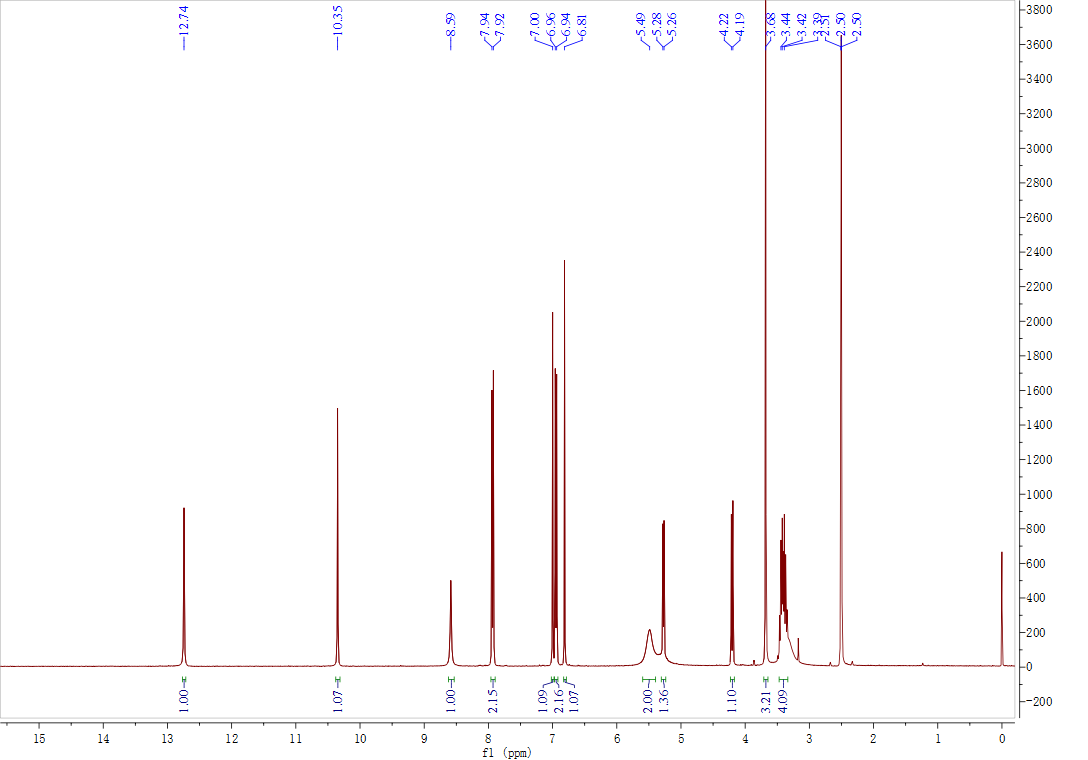


**Figure S6.** ^1^H-NMR spectra of Scu-Me

**Figure S7.** Mass spectra of Scu-Me-C5-TG


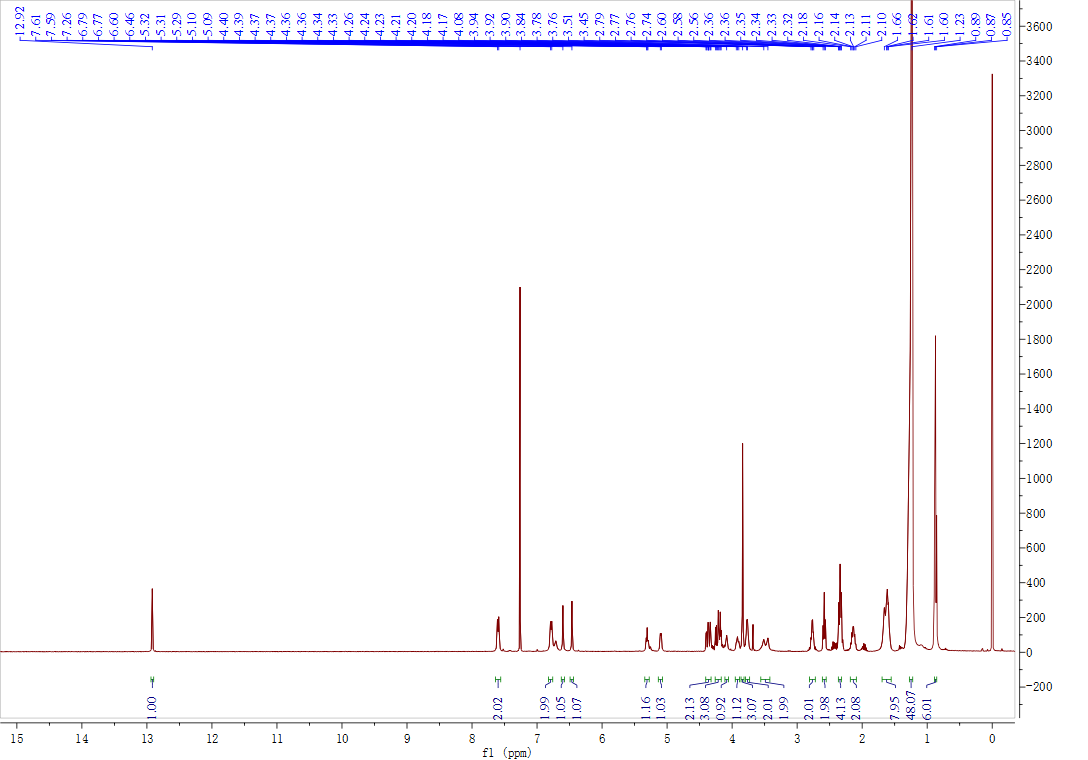


**Figure S8.** ^1^H-NMR spectra of Scu-Me-C5-TG


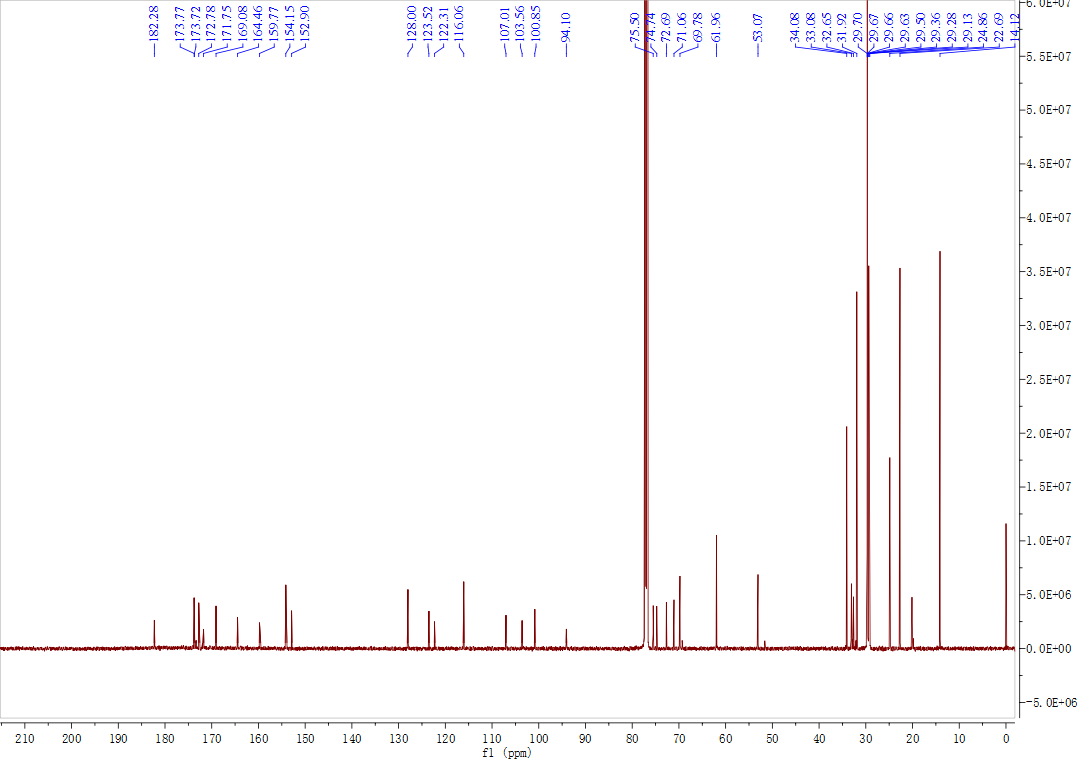


**Figure S9.** ^13^C-NMR spectra of Scu-Me-C5-TG

**Figure S10.** Mass spectra of Scu-Me-C5-βMe-TG


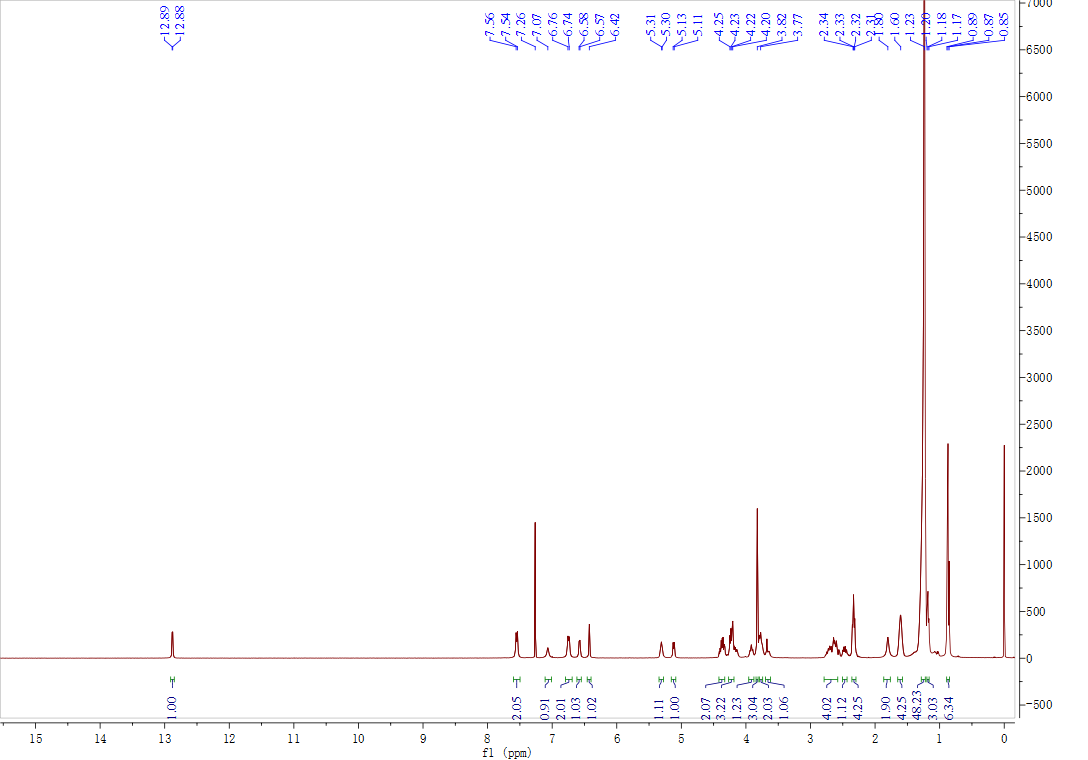


**Figure S11.** ^1^H-NMR spectra of Scu-Me-C5-βMe-TG


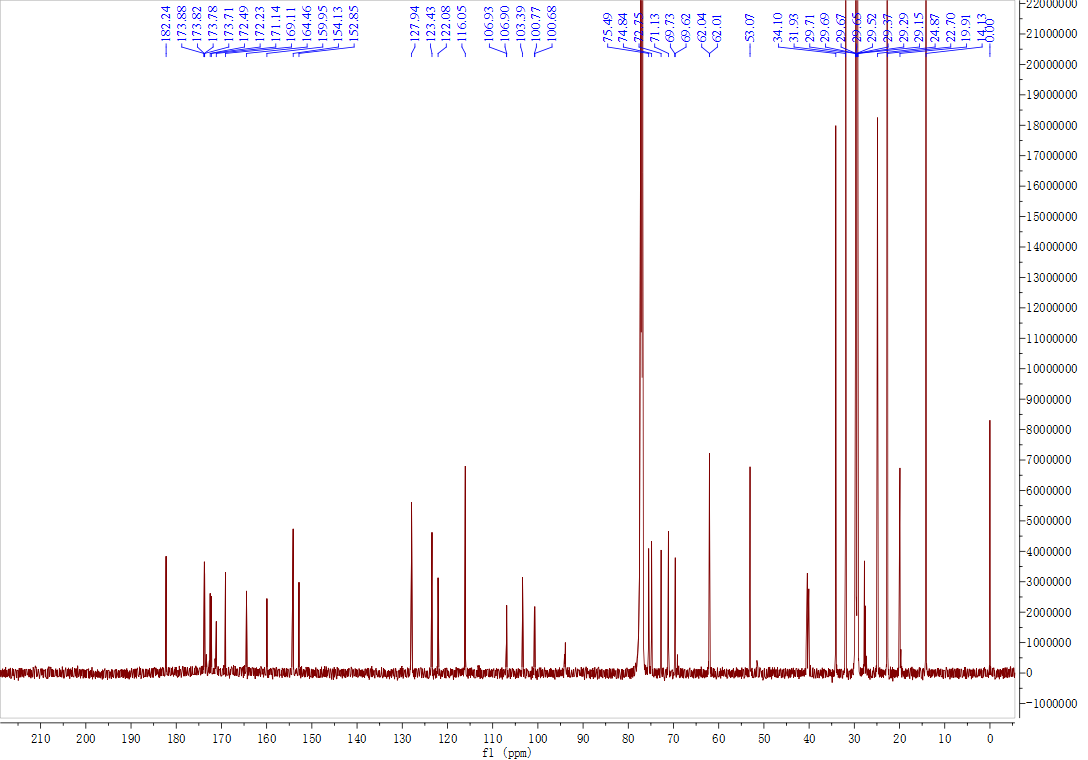


**Figure S12.** ^13^C-NMR spectra of Scu-Me-C5-βMe-TG

1. **Mass spectra of in vitro and in vivo samples**


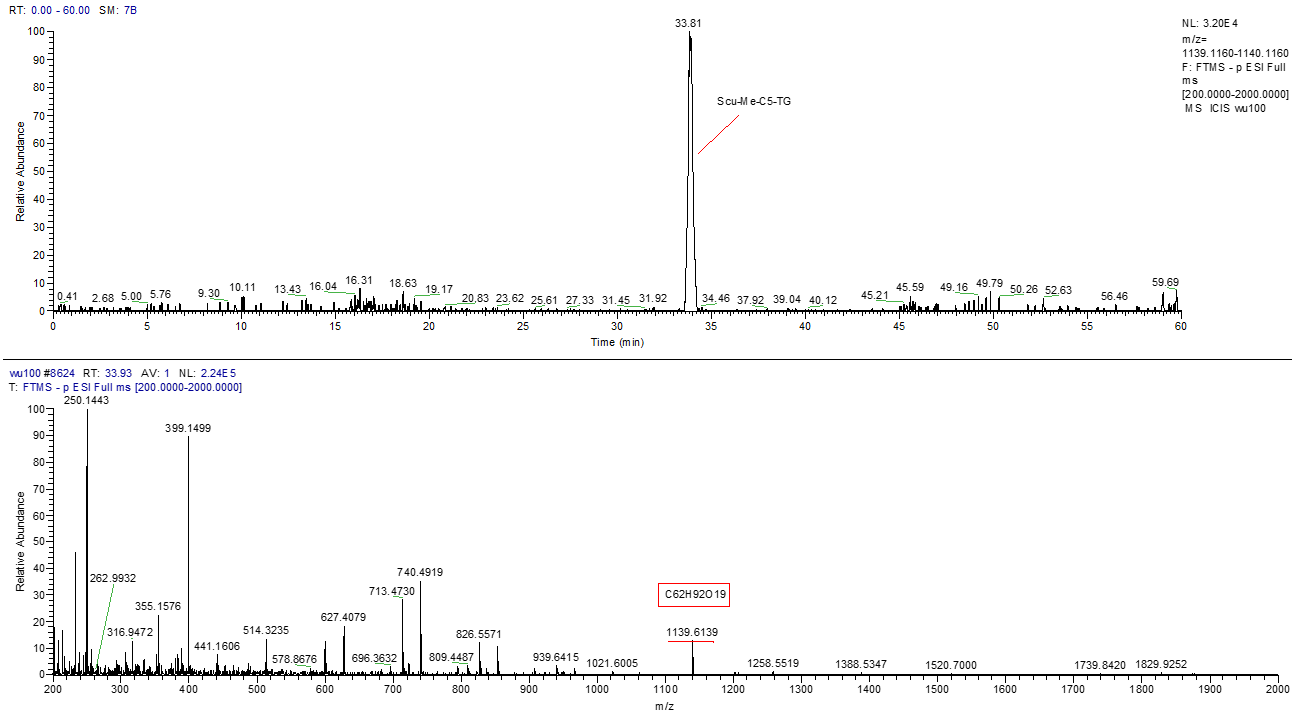


**Figure S13.** Mass spectrometric test results of Scu-Me-C5-TG samples in vitro


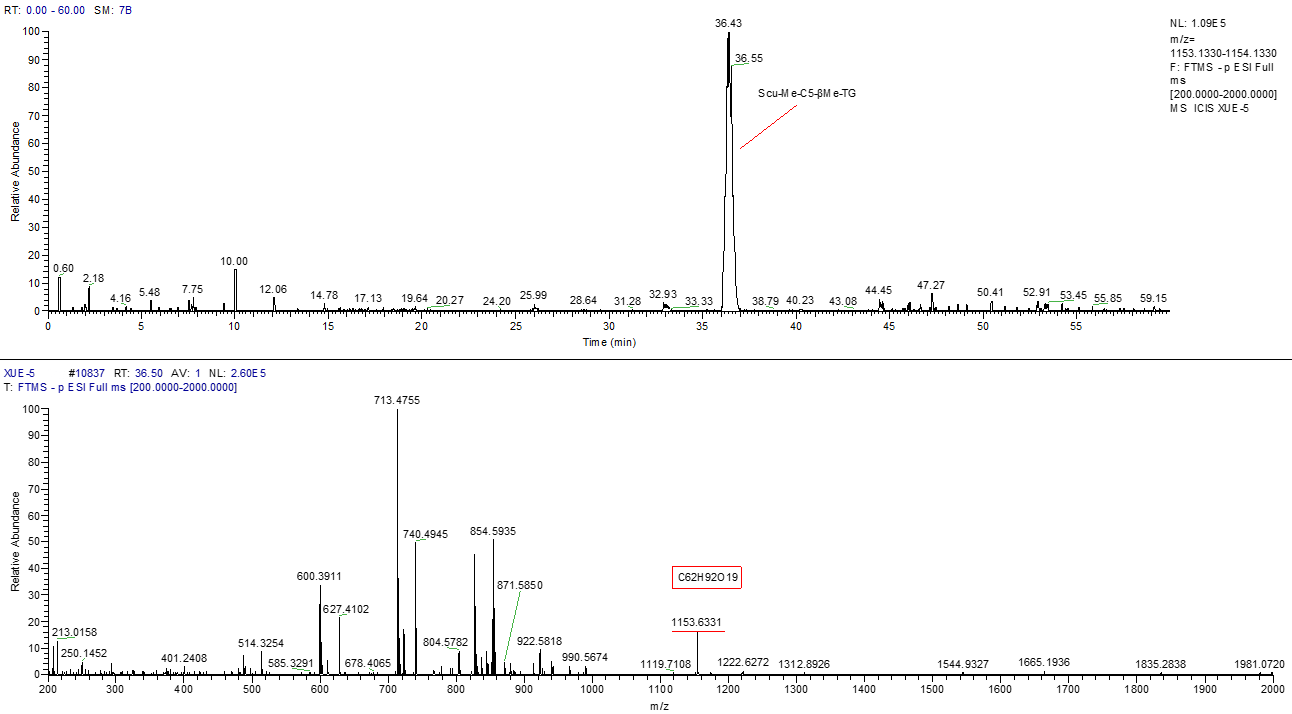


**Figure S14.** Mass spectrometric test results of Scu-Me-C5-βMe-TG samples in vitro

**Figure S15.** Mass spectra of the collected mesenteric lymph


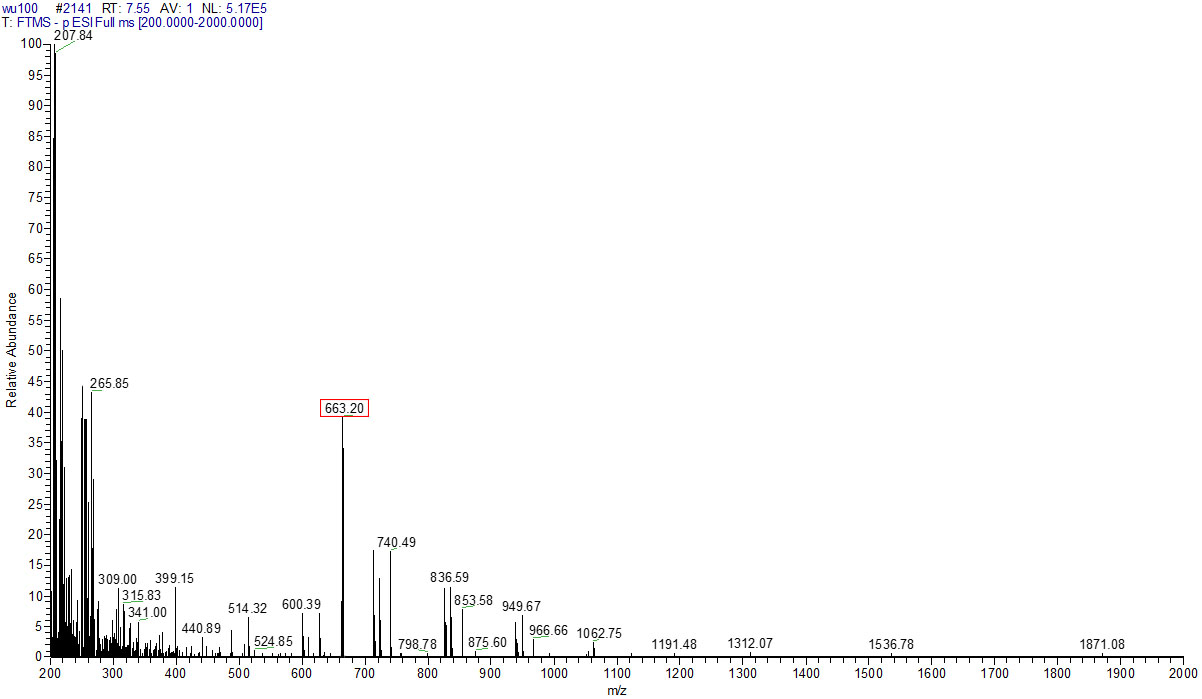


**Figure S16.** Mass spectra of Scu-Me-C5-MG


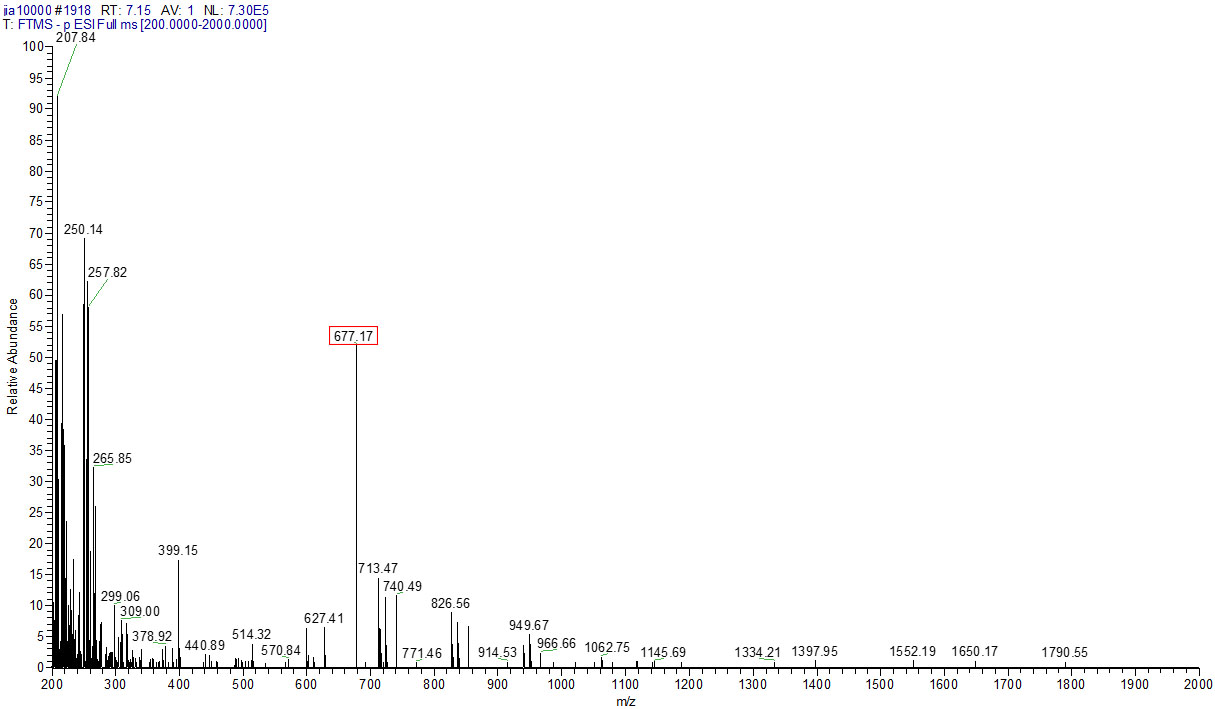


**Figure S17.** Mass spectra of Scu-Me-C5-βMe-MG


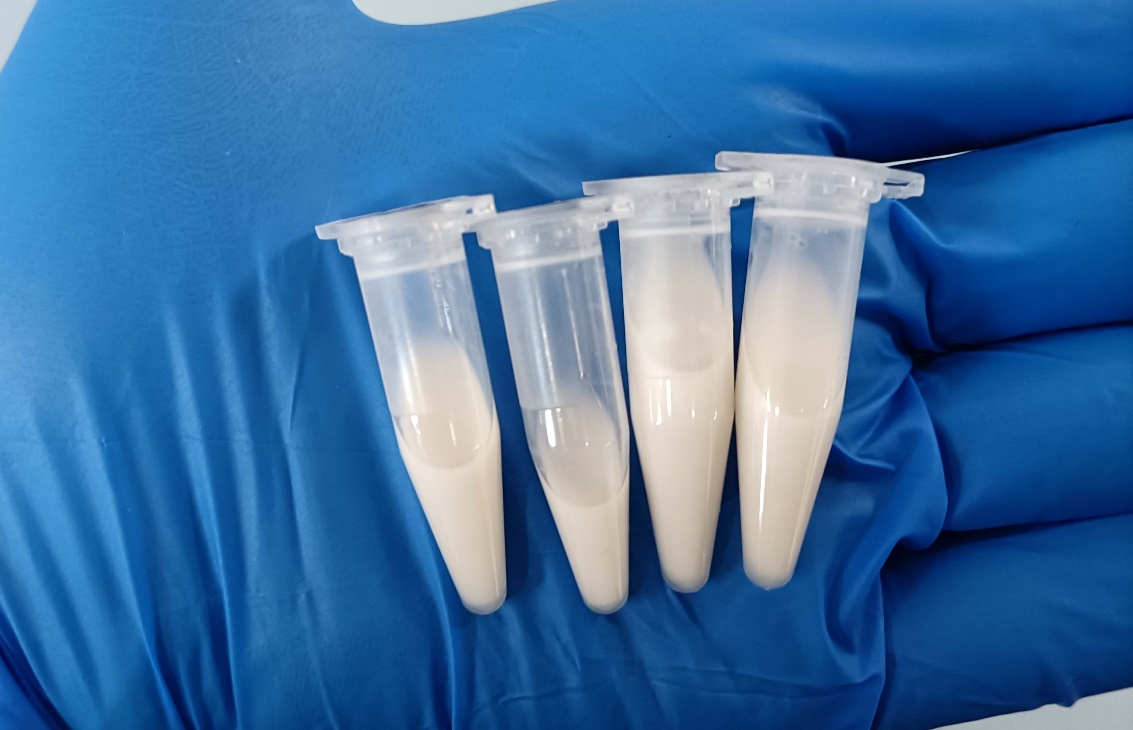


**Figure S18.** A portion of the collected mesenteric lymph
